# MDA5 variants trade antiviral activity for protection from autoimmune disease

**DOI:** 10.1186/s12920-025-02171-y

**Published:** 2025-06-02

**Authors:** Chris Wallace, Rahul Singh, Yorgo Modis

**Affiliations:** 1 Cambridge Institute of Therapeutic Immunology and Infectious Disease, University of Cambridge School of Clinical Medicine, Cambridge, UK; 2 MRC Biostatistics Unit, University of Cambridge, Cambridge, UK; 3 Molecular Immunity Unit, Department of Medicine, University of Cambridge, MRC Laboratory of Molecular Biology, Francis Crick Avenue, Cambridge, UK

**Keywords:** Type 1 diabetes (T1D), Inflammatory bowel disease (IBD), Human genetic variants, Genome-wide association studies (GWAS), Fitness trade-off, Autoimmune disease

## Abstract

Loss-of-function variants in MDA5, a key sensor of double-stranded RNA from viruses and retroelements, have been associated with protection from type 1 diabetes (T1D) in genome-wide association studies (GWAS). MDA5 loss-of-function variants have also been reported to increase the risk of inflammatory bowel disease (IBD). Whether these associations are linked or extend to other diseases remains unclear. Here, fine-mapping analysis of four large GWAS datasets shows that T1D-protective loss-of-function MDA5 variants also protect against psoriasis and hypothyroidism, while increasing the risk of IBD. The degree of autoimmune protection and IBD risk were linearly proportional. The magnitudes of the odds ratios for autoimmune protection and IBD risk were larger for rare MDA5 variants than for common variants, which were differentially expressed in different geographic populations. Our analysis suggests MDA5 genetic variants offer a direct fitness trade-off between viral clearance and autoimmune tissue damage.

**Supplementary Information:**

The online version contains supplementary material available at 10.1186/s12920-025-02171-y.

## Introduction

Innate immune responses must be sensitive enough to detect infection yet specific enough to avoid activation by cellular components. A key innate immune receptor for cytosolic double-stranded RNA (dsRNA), a potent signature of viral infection, is MDA5 [1–5]. Recognition of dsRNA by MDA5 induces a potent IFN-β response [2, 3]. The ATPase activity and dsRNA binding cooperativity of MDA5 confer the necessary sensitivity and specificity of MDA5-dsRNA recognition [4–6]. Mutations in the gene encoding MDA5, *IFIH1*, can perturb the balance between sensitive and specific dsRNA recognition. *IFIH1* is a hotspot for natural variants with clinical associations. Approximately 40 gain-of-function missense variants have been associated with interferonopathies [7–9]. These variants promote formation of MDA5 signaling complexes, including on endogenous dsRNAs, by either increasing the RNA binding affinity of MDA5 or inhibiting its ATP-dependent proofreading activity [5, 9, 10]. Loss-of-function MDA5 variants cause recurrent infections [11], and have been reported to contribute to inflammatory bowel disease (IBD), including ulcerative colitis and Crohn’s disease [12, 13]. GWAS data have also associated loss-of-function MDA5 variants with reduced risk of developing certain autoimmune diseases, most notably T1D [14–19]. Specifically, variants E627* (rs35744605), R843H (rs3747517), I923V (rs35667974), and T946A (rs1990760) have been identified as T1D-protective [14–18, 20, 21]. The E627* and I923V variants are rare, with allele frequencies of 1–2%, while R843H and T946A are common. These common variants are differentially expressed in different geographic populations. The T946A variant is present in 70–80% of Africans and Asians but only 30–50% of Caucasians [15–18, 20]. Similarly, the R843H variant is found in 70% of Asians but only 30–40% of Caucasians and Africans [15–18, 20]. Most human *IFIH1* reference sequences contain the alleles that are most common in Asians (Ala946/His843). These coding variants inhibit the formation of MDA5-dsRNA signaling complexes [18, 22] by reducing the RNA binding affinity of MDA5 or hyperactivating its ATPase activity [23]. Other loss-of-function variants are splice donor variants that reduce splicing efficiency and hence mRNA levels [19].

A robust clinical link has emerged between T1D onset and recent infection with RNA viruses, in particular coxsackieviruses and other enteroviruses [24]. Patients with T1D have more frequent and persistent enterovirus infections, which precede the appearance of prediabetic markers, including autoantibodies [25]. MDA5 recognizes RNA from *Picornaviridae*, including enteroviruses [1, 26], which have evolved mechanisms to suppress IFN-β transcription [27, 28]. MDA5-induced inflammation in the pancreas following rotavirus infection contributes to autoimmune destruction of pancreatic β-cells [29]. Therefore, a plausible hypothesis is that MDA5-dependent inflammation following viral infection can trigger autoimmune β-cell killing.

Gastrointestinal viral infection has also been linked to IBD onset [30]. Intestinal cells from patients with IBD harboring loss-of-function MDA5 variants had elevated viral loads and were compromised in their ability to maintain epithelial barrier integrity upon further exposure to the enteric virome [13]. Hence, the frequency and clinical phenotypes of MDA5 loss-of-function variants in patients with IBD suggest that MDA5 deficiency contributes to the induction of IBD [12, 13], due in part to increased exposure and susceptibility to viral infection [11, 13].

## Results and discussion

**Fig. 1:**
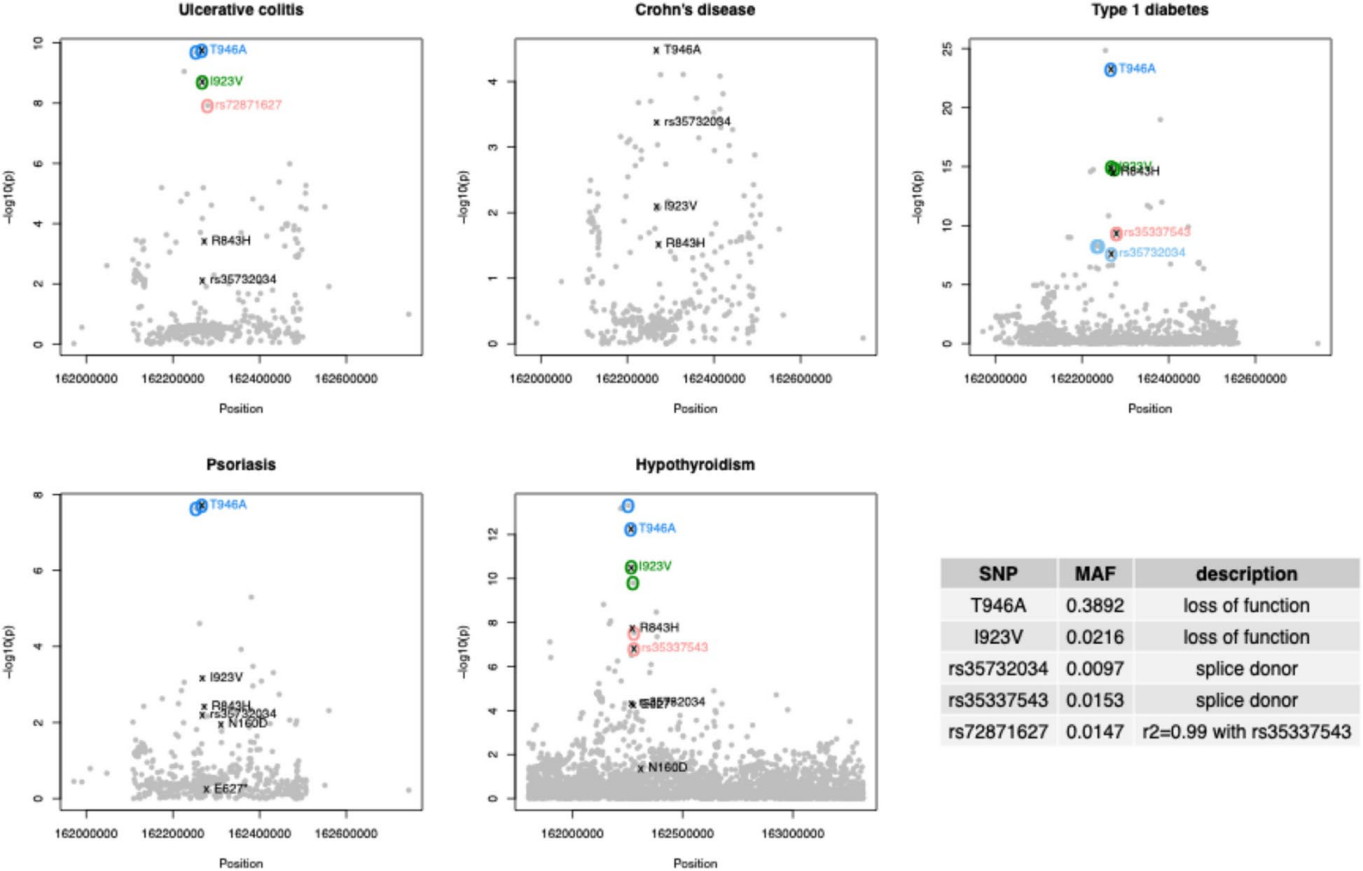
Manhattan plots of fine-mapped variants for five diseases. Colored circles indicate the fine mapping sets identified, and colored text labels the most likely causal SNP in each set, selected due its known function on MDA5. Known loss-of-function SNPs that were not in any fine mapping set are marked with “x” and a black label. Lower right, minor allele frequency (MAF) and function of fine-mapped SNPs

Loss-of-function MDA5 variants have been associated with recurrent infection [11], T1D protection [14–19], and IBD risk [12, 13]. However, whether these associations are linked, or whether they apply to autoimmune disease more broadly, has not been explored. We addressed these questions by utilizing larger, more recent GWAS studies to conduct a broader, more sensitive survey of immune-mediated disease associations involving MDA5 variants. For our analysis, we selected GWAS datasets to maximize the likelihood of accurate fine mapping – balancing the need for large sample size, minimizing the use of imputed genotypes, and using ancestries that best matched the reference panel used to derive linkage disequilibrium (LD) estimates [31–34] (see Methods). By reviewing associations with a reported lead T1D-associated variant, I923V (rs35667974) [15] and associations with *IFIH1* reported by the Open Targets Platform (https://platform.opentargets.org/target/ENSG00000115267/associations) we identified psoriasis, hypothyroidism, Crohn’s disease, and ulcerative colitis as additional associated diseases with suitable and available GWAS summary statistics. Fine-mapping analysis using GWAS summary data for these five diseases identified four variants likely to be causally associated with a subset of the diseases, with considerable overlap of variants between diseases (Fig. 1**, **Table 1). Two of these variants were coding variants, I923V and T946A, and two were splice donor variants, rs35337543 and rs35732034. All four variants were previously associated with T1D protection [14–19]. Notably, fine mapped signals indexed by each of these variants showed strong evidence of colocalization across diseases (posterior probabilities > 0.98, see Table 2 and Additional File 1).

**Table 1:** Single variant association and fine-mapping analysis results

Variant	SNP	Location	Allele	EAF	Disease	lnOR ± std. err	OR	P	P_Bonferroni_^b^	Causal^c^
I923 V	rs35667974	2:162,268,127	T > C	0.022	T1D	−0.493 ± 0.062	0.611	1.21** × **10^–15^	3.51** × **10^–14^	T
					Psoriasis	−0.440 ± 0.129	0.644	6.68** × **10^–4^	0.0194	F
					Hypothyr	−0.254 ± 0.038	0.776	2.98** × **10^–11^	8.65** × **10^–10^	T
					Crohn’s	0.121 ± 0.046	1.129	8.08** × **10^–3^	0.2342	F
					UC	0.276 ± 0.046	1.318	1.94** × **10^–9^	5.61** × **10^–8^	T
Splice1	rs35732034	2:162,268,086	C > T	0.010	Psoriasis	−0.693 ± 0.253	0.500	6.25** × **10^–3^	0.1812	F
					T1D	−0.464 ± 0.083	0.629	2.29** × **10^–8^	6.64** × **10^–7^	T
					Hypothyr	−0.230 ± 0.056	0.794	4.47** × **10^–5^	1.30** × **10^–3^	F
					UC	0.180 ± 0.067	1.197	7.70** × **10^–3^	0.2233	F
					Crohn’s	0.226 ± 0.064	1.253	4.15** × **10^–4^	0.0120	F
Splice2^a^	rs72871627	2:162,280,432	A > G	0.015	Psoriasis	−0.541 ± 0.200	0.582	6.81** × **10^–3^	0.1975	F
					T1D	−0.458 ± 0.073	0.633	4.19** × **10^–10^	1.21** × **10^–8^	T
					Hypothyr	−0.253 ± 0.046	0.777	2.94** × **10^–8^	8.52** × **10^–7^	T
					Crohn’s	0.072 ± 0.056	1.074	0.1990	1	F
					UC	0.308 ± 0.054	1.360	1.19** × **10^–8^	3.45** × **10^–7^	T
Splice2	rs35337543	2:162,279,995	C > G	0.015	T1D	−0.458 ± 0.073	0.633	4.19** × **10^–10^	ND	T
					Hypothyr	−0.236 ± 0.045	0.790	1.51** × **10^–7^	ND	T
E627^a^	rs35744605	2:162,277,580	C > A	0.007	Hypothyr	−0.273 ± 0.068	0.761	5.63** × **10^–5^	1.63** × **10^–3^	F
					Psoriasis	−0.210 ± 0.372	0.810	0.5717	1	F
T946 A	rs1990760	2:162,267,541	T > C	0.389	Psoriasis	−0.192 ± 0.034	0.825	1.95** × **10^–8^	5.66** × **10^–7^	T
					T1D	−0.132 ± 0.013	0.876	5.57** × **10^–24^	1.61** × **10^–22^	T
					Hypothyr	−0.080 ± 0.011	0.923	5.61** × **10^–13^	1.63** × **10^–11^	T
					Crohn’s	0.053 ± 0.013	1.055	3.36** × **10^–5^	9.75** × **10^–4^	F
					UC	0.086 ± 0.013	1.089	1.78** × **10^–10^	5.16** × **10^–9^	T
R843H	rs3747517	2:162,272,314	C > T	0.276	T1D	−0.111 ± 0.014	0.895	2.94** × **10^–15^	8.51** × **10^–14^	F
					Psoriasis	−0.103 ± 0.035	0.903	3.72** × **10^–3^	0.1079	F
					Hypothyr	−0.068 ± 0.012	0.934	1.85** × **10^–8^	5.37** × **10^–7^	F
					Crohn’s	0.030 ± 0.014	1.031	0.0301	0.8730	F
					UC	0.051 ± 0.014	1.053	3.85** × **10^–4^	0.0112	F
N160D	rs74162075	2–162,310,909	T > C	0.001	Psoriasis	−2.057 ± 0.811	0.128	0.0111	0.3231	F
					Hypothyr	−0.270 ± 0.133	0.763	0.0427	1	F

Dashed lines indicate that the SNPs above and below the line are in LD with each other

*EAF* effect allele frequency, *OR* odds ratio, *ND* Not done

^a^rs72871627 was used as a tag of rs35337543 because the latter was not available in all GWAS datasets

^b^P_Bonferroni_ was adjusted for 29 tests

^c^Causality was inferred from presence of the SNP in a credible set in fine mapping analysis

**Table 2:** Colocalization analysis of fine mapped signals

d1	d2	Lead SNP d1	Lead SNP d2	N. SNPs	PP.H0.abf	PP.H1.abf	PP.H2.abf	PP.H3.abf	PP.H4.abf
UC	T1D	T946 A	T946 A	388	5.35** × **10^–24^	2.66** × **10^–21^	7.14** × **10^–6^	1.55** × **10^–3^	0.9984
UC	Psoriasis	T946 A	T946 A	379	5.93** × **10^–08^	2.94** × **10^–5^	8.23** × **10^–6^	2.09** × **10^–3^	0.9979
T1D	Psoriasis	T946 A	T946 A	460	1.98** × **10^–23^	2.64** × **10^–5^	2.75** × **10^–21^	1.68** × **10^–3^	0.9983
UC	Hypothyr	T946 A	T946 A	405	2.37** × **10^–11^	1.18** × **10^–8^	9.57** × **10^–6^	2.76** × **10^–3^	0.9972
T1D	Hypothyr	T946 A	T946 A	1150	2.79** × **10^–26^	3.72** × **10^–8^	1.13** × **10^–20^	0.01308	0.9869
UC	T1D	I923 V	I923 V	388	9.59** × **10^–8^	5.58** × **10^–7^	3.46** × **10^–4^	9.09** × **10^–6^	0.9996
UC	Hypothyr	I923 V	I923 V	405	1.03** × **10^–5^	5.97** × **10^–5^	3.47** × **10^–4^	1.09** × **10^–5^	0.9996
T1D	Hypothyr	I923 V	I923 V	1150	1.52** × **10^–8^	7.79** × **10^–5^	6.15** × **10^–7^	1.16** × **10^–3^	0.9988
UC	T1D	rs72871627	rs72871627	388	3.97** × **10^–14^	1.93** × **10^–10^	4.11** × **10^–7^	1.33** × **10^–6^	0.9999
UC	Hypothyr	rs72871627	rs72871627	405	1.23** × **10^–10^	6.00** × **10^–7^	4.11** × **10^–7^	1.34** × **10^–6^	0.9999
T1D	Hypothyr	rs72871627	rs72871627	1150	5.01** × **10^–14^	1.01** × **10^–6^	1.94** × **10^–10^	1.92** × **10^–3^	0.9981

d1 and d2 are the disease datasets in use

Lead SNP d1 and Lead SNP d2 index the signals used

N. SNPs shows the number of SNPs used in the test

PP.H*i*.abf values (columns 6–10) show the posterior probability of colocalization hypotheses H0..H4. The posterior is always concentrated in PP.H4.abf, which is the hypothesis of a shared causal variant

Given the overlaps between diseases, and the small *P* values observed for fine-mapped variants in one disease when considering another, we called associated variants by considering single variant association at any of these variants or other known loss-of-function variants not in LD with any of the index variants using a stringent Bonferroni correction. This identified associations of a fifth variant, E627* (rs35744605), with psoriasis, and hypothyroidism (Fig. 1). While T946A is common, the other variants are rare (allele frequency below 3%). Fine-mapping analysis did not find evidence for causality for variants E627* and R843H (Table 1). However, for E627* the lack of evidence of causality is likely due to its low allele frequency (0.7%), which limits power in genetics studies. Association of R843H with T1D protection is likely attributable to LD with the co-occurring causal T946A allele (*r*^2^ = 0.60), as previously reported [15, 16, 18]. Our accompanying biochemical and structural study of T1D-protective MDA5 variants shows that the E627* variant lacks signaling activity due to a loss of RNA binding affinity, confirming that this variant leads to complete loss of MDA5 function. In contrast, neither the T946A variant nor the R843H variant had any significant effects on MDA5-dependent interferon expression in response to picornavirus infection, leaving underlying mechanism of disease association for these variants unclear. We hypothesize that the T946A variant may be causal through loss of threonine phosphorylation, but in the absence biochemical data explaining how T946A causes disease we have also included the R843H variant in this study [23].

**Fig. 2:**
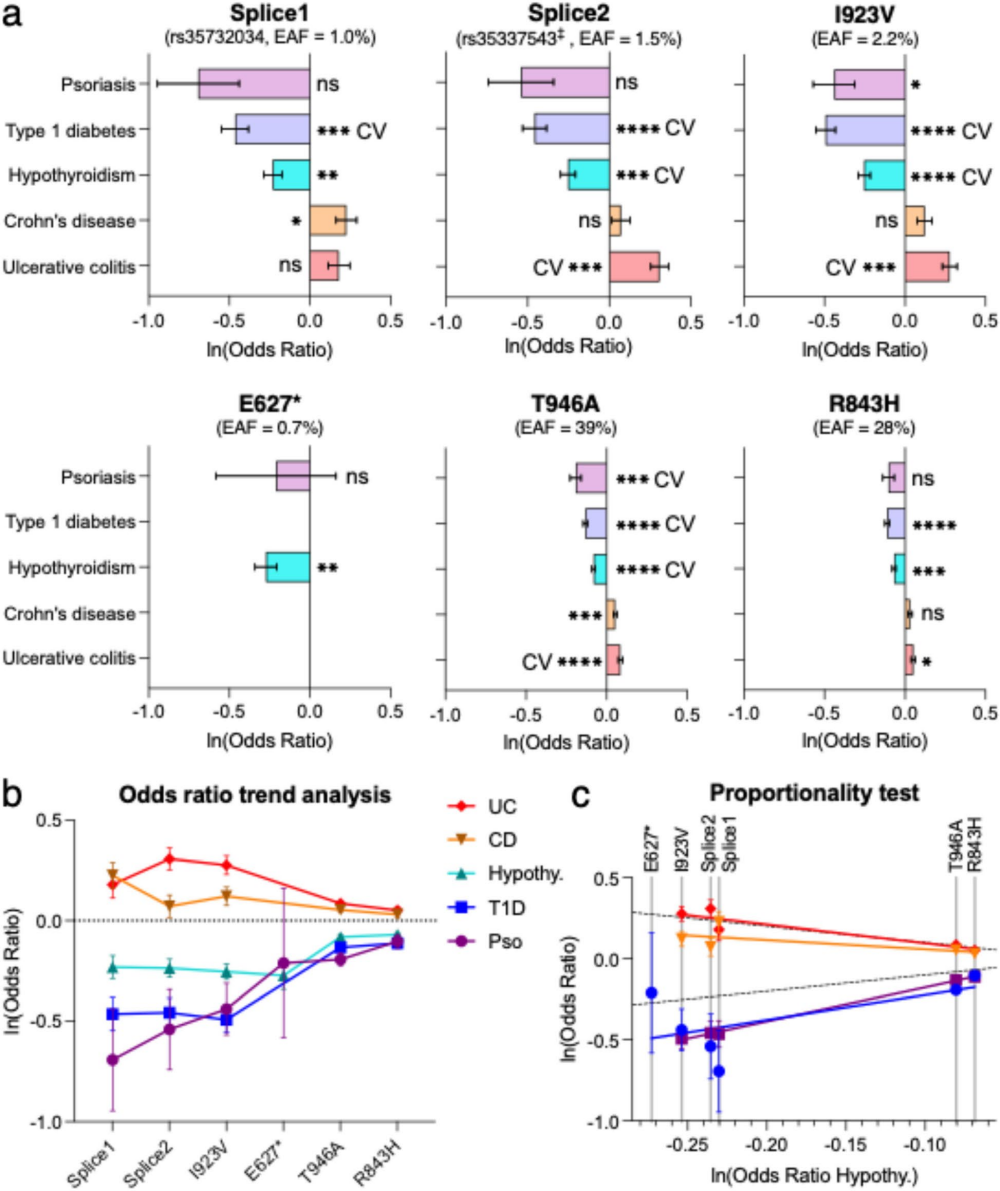
GWAS meta-analysis of loss-of-function MDA5 variants. **a** Effect sizes expressed in terms of the odd ratio for selected MDA5 loss-of-function variants. ^‡^Because rs35337543 was only available in two GWAS datasets, rs72871627 (*r*^2^ = 0.973) was used as a tag for rs35337543. EAF, effect allele frequency. Error bars, SEM. ns, *P* > 0.05; *, *P* < 0.05; **, *P* < 0.01; ***, *P* < 0.001; ****, *P* < 5** × **10^–8^ (genome-wide significance), where P is the Bonferroni-adjusted *P* value from single variant association analysis. CV, likely causal variant present in a credible set in fine-mapping analysis. See Table 1. **b** Odds ratio plot for variants and diseases in *(a)*. **c** Estimated effect sizes in hypothyroidism compared to the other diseases. Hypothyroidism was selected because it was the largest dataset for which all SNPs were available. Dashed lines, identity and negative identity lines

Comparing associations across diseases, we found that all five associated variants offered protection against T1D, psoriasis and hypothyroidism, but increased the risk of ulcerative colitis and Crohn’s disease (Fig. 2a). The significance of the associations varied across diseases, with T1D and hypothyroidism showing the highest significance, whereas Crohn’s disease and psoriasis showed marginal significance for some variants. Remarkably, however, a strict correlation was observed between protection and risk (Fig. 2b). A statistical test of proportionality (see Methods) concluded that changes in risk were linearly proportional across variants (Fig. 2c). Additionally, the magnitudes of the odds ratios were larger for the rare variants than for the common variants. Thus, the rarest variant, I923V, conferred the greatest degree of T1D protection and the greatest risk of IBD, whereas the most common variant, T946A, was associated with the least T1D protection and IBD risk (Fig. 2a-b). Notably, the T946A variant, while common in all populations, is the major allele in Asian and African populations but the minor allele in Caucasians.

There are some limitations to our study. Given variable allele frequencies of T946A between ancestries and the importance of confirming findings in diverse populations, it would be worthwhile to repeat this analysis in non-European populations. However, fine mapping requires large GWAS samples to have the power to distinguish between variants, and the only such studies we could find were in European populations. Additionally, even with the available large European GWAS datasets, the low allele frequency of certain variants, particularly as E627* and N160D, limited the power of our meta-analyses and likely prevented causal effects from being detected.

Our GWAS meta-analysis uncovers a direct correlation between protection from autoimmune-related diseases (T1D, psoriasis and hypothyroidism) and increased risk of IBD. The implication is that calibration of MDA5-dependent antiviral signaling offers a fundamental fitness trade-off. Loss-of-function MDA5 variants protect from autoimmune tissue damage, including to pancreatic β-cells leading to T1D, but increase the risk of inflammatory tissue damage from persistent infection, including by enteric viruses in intestinal epithelia leading to IBD. This model predicts that gain-of-function MDA5 variants protect from chronic inflammation and IBD by ensuring infections are cleared but do so at the cost of increasing the risk of T1D and other autoimmune diseases. The clinical consequences and fitness trade-offs of MDA5 variants have implications for treatment and prevention of infectious, inflammatory, and autoimmune disease. For example, T1D risk scores and doses of RNA-based vaccines could be adjusted accordingly for patients with gain- or loss-of-function MDA5 variants. As a general sensor of elevated dsRNA resulting from infection or environmental stress, MDA5 is an attractive target for immunomodulatory therapies for cancer and autoinflammatory disorders. Any interventions targeting MDA5 would benefit from dosage optimization based on the patient’s *IFIH1* genotype, which will vary according to geographic or ethnic origin, and the single variant associations reported here.

## Methods

### GWAS meta-analysis

GWAS summary data for a reported lead T1D-associated variant, I923V (rs35667974) were downloaded for the following diseases: T1D (16,000 T1D cases, 25,000 controls) [31]; IBD (5,956 Crohn’s disease cases, 6,968 ulcerative colitis cases, 21,770 controls) [32]; psoriasis (19,032 cases, 286,769 controls) [33]; and hypothyroidism (20,563 cases, 399,910 controls) [34]. We prioritized ImmunoChip datasets where available, because the uniform coverage over SNPs is likely to produce more accurate fine mapping results than in (possibly larger) GWAS meta analyses [35]. Data were downloaded from the GWAS catalog from the following repositories: ulcerative colitis, https://ftp.ebi.ac.uk/pub/databases/gwas/summary_statistics/GCST003001-GCST004000/GCST003045/harmonised/26192919-GCST003045-EFO_0000729.h.tsv.gz (ImmunoChip); Crohn’s disease, https://ftp.ebi.ac.uk/pub/databases/gwas/summary_statistics/GCST003001-GCST004000/GCST003044/harmonised/26192919-GCST003044-EFO_0000384.h.tsv.gz (ImmunoChip); T1D, https://ftp.ebi.ac.uk/pub/databases/gwas/summary_statistics/GCST90013001-GCST90014000/GCST90013445/GCST90013445_buildGRCh38.tsv (ImmunoChip); Psoriasis: http://ftp.ebi.ac.uk/pub/databases/gwas/summary_statistics/GCST005001-GCST006000/GCST005527/harmonised/23143594-GCST005527-EFO_0000676.h.tsv.gz (Meta analysis); Hypothyroidism: https://pan-ukb-us-east-1.s3.amazonaws.com/sumstats_flat_files/categorical-20002-both_sexes-1453.tsv.bgz (genome wide SNP chip). These studies were selected to balance the need for large studies and those most likely to give accurate results in fine mapping – i.e. those which minimized imputation and most closely matched the ancestry of our reference LD panel.

Alleles were aligned to the UK Biobank as a common reference. Reference LD matrices were estimated from 40,000 European subjects from UK Biobank. Fine mapping was performed with a variant of the Sum of Single Effects (SuSiE) [36] model, as implemented in susieR [37]. We identified distinct signals as those tagged by 95% credible sets in each analysis.

rs72871627 was selected as a tag (*r*^2^ = 0.99) of the splice donor variant rs35337543 because rs72871627 was available in all GWAS datasets whereas rs35337543 was only available in the T1D GWAS dataset.

Tests of colocalization of individual fine mapped signals were performed using coloc.susie [38] with default parameters. Tests of proportional effects between pairs of diseases were performed across all available variants shown in Fig. 2c using colocPropTest (https://cran.r-project.org/package=colocPropTest) which implements the test of proportionality as previously described [39].

## Supplementary Information

Additional file 1. Detailed fine mapping results for the five diseases analyzed. Each tab shows the fine mapping results for a single disease. The first column lists the chromosomal location and allele of the SNP. The second column shows the posterior inclusion probability (PIP) for the SNP, i.e. the probability this SNP is causal – these probabilities may sum more than 1 over SNPs if there are more than one credible sets. The remaining columns show the posterior probabilities (PP) that the SNP are in the *i*th credible set, with one column per credible set detected by SuSiE [36–38]. When there is only one credible set then the PIP equals the posterior probability for that credible set; when there is more than one credible set, the PIP is a summary measure over all sets.

## Data Availability

This study is a meta-analysis of previously published data. All code to reproduce this analysis is available at Zenodo.org (10.5281/zenodo.12771481).

## Abbreviations

ATP: Adenosine triphosphate
GWAS: Genome-wide association study
IBD: Inflammatory bowel disease
LD: Linkage disequilibrium
MDA5: Melanoma differentiation-associated protein 5
SNP: Single nucleotide polymorphism
T1D: Type 1 diabetes
